# Should new health technology be available only for patients able and willing to pay?

**DOI:** 10.1080/20016689.2017.1315294

**Published:** 2017-05-04

**Authors:** Piet Calcoen, Albert Boer, Wynand P.M.M. van de Ven

**Affiliations:** ^a^ Department of Health Policy and Management, Erasmus University Rotterdam, Rotterdam, The Netherlands

**Keywords:** Health technology, accessibility, health insurance, out-of-pocket payments, choice, transparency

## Abstract

New health technology comes on the market at a rapid pace and – sometimes – at a huge cost. Providing access to new health technology is a serious challenge for many countries with mandatory health insurance.

This article analyses access to new health technology in Belgium and the Netherlands, using eight concrete examples as a starting point for comparing the two – neighbouring – countries. Contrary to the Netherlands, out-of-pocket payments for new health technology are widely accepted and practiced in Belgium. This difference is largely the result of different regulatory environments. A major difference is the way that entitlements to care are described: closed and explicit in Belgium versus open and non-explicit in the Netherlands. The characteristics of in-kind policies versus reimbursement policies also play a role.

Allowing out-of-pocket payments for new health technology has consequences for the patients. It leads to greater access to new health technology (for those who are able and willing to pay), but has a negative effect on equal access to care. Choice and transparency are enhanced by allowing out-of-pocket payments for new health technology.

It could be argued that lack of coverage by mandatory health insurance should not render private access to new health technology impossible.

## Introduction

In many countries with mandatory health insurance, a serious challenge is how to deal with new health technology, for example, an innovative hip prosthesis, a computerized prosthetic leg, robot-assisted cardiac surgery, non-invasive prenatal testing and new cancer medicines and orphan drugs. While mandatory health insurance generally covers a broad range of health technology, new technology may not be – readily – covered because of budgetary reasons or because there is no unanimity (yet) about the evidence-based character or the medical necessity. In case of doubt, national health authorities can decide not to cover a new health technology, even if the technology has been acknowledged by health technology assessment centres and/or is covered by health insurers in other countries.

Should new health technologies – that have proved at least an acceptable level of evidence–be available for all, possibly with some cost-sharing, or only for those who are able and willing to pay the full cost?

Using two neighbouring countries (Belgium and the Netherlands) as case studies, we will discuss and analyse different options for policy-makers to deal with new health technology. In Belgian hospitals there are lists with out-of-pocket payments for well-defined health technologies available for the patient [1,2]. Whereas standard treatment A is covered by mandatory basic health insurance, for treatment B, applying new health technology, one must pay the listed additional out-of-pocket payments. For examples of such treatments B in Belgium, see Table 1.

**Table 1.** Health technology available to patients but to be financed out-of-pocket (Belgium, 2015).

New health technology	Price (to be paid out-of-pocket)
Robot-assisted coronary bypass surgery (da Vinci) [3]	€1,200
Trabecular metal acetabular revision system (Zimmer) (revision hip replacement surgery) (2011–2014, 4]	€2,569
Cervical intervertebral disc prosthesis (cervical degenerative disc disease or herniated disc) [1]	€2,776
Microprocessor-controlled prosthetic leg (Genium) [3]	€27,177
MammaPrint (gene assay for breast cancer patients) [3,5]	€2,675
Non-invasive prenatal testing (NIPT) (serum marker screening for certain chromosomal abnormalities in a developing foetus) [1]	€460
Ofatumumab (Arzerra) 300 mg + (7*1000 mg) or 300 mg + (11*2000 mg) (orphan drug to treat chronic lymphocytic leukaemia) [6–8]	€17,875–€54,604
Nivolumab (Opdivo) 3 mg/kg every two weeks (6 months treatment) (cancer medicine to treat adults with melanoma or lung cancer) [6–8]	€48,972

In Belgium there is an enumerative, limitative list of medical goods and services covered by mandatory health insurance (Dutch: ‘nomenclatuur van de geneeskundige verstrekkingen’). Whether a new technology gets on this list is being decided by the national health authorities. Reimbursement can be denied when national authorities find there may be an issue of therapeutic evidence or cost-effectiveness. In the Netherlands, there is no such list. New technology is covered by mandatory health insurance if it meets the criteria of ‘current scientific knowledge and practice’ (Dutch: ‘stand van wetenschap en praktijk’).

The listed treatments B, as well as the additional out-of-pocket payments, may differ among hospitals. Only patients who are able and willing to pay the additional out-of-pocket payments have access to treatment B. People can buy voluntary additional insurance that covers these additional out-of-pocket expenses.

In the Netherlands the situation is totally different. Dutch hospitals do not have such lists and the general perception is that additional payments for new health technology are legally forbidden. So, at first glance, the Belgian health care system could be characterized as a two-tier system and the Dutch health care system as an egalitarian system.

This observation raises several questions. First, are these new health technologies in the Netherlands available for all, for nobody, or only for selected groups of patients? In the latter case: for which groups, and are the selection criteria explicit and transparent? Second, how can the observed differences between Belgium and the Netherlands be explained? Third, what are the consequences for the patient?

The goal of this article is to answer the above-mentioned questions. According to the phrase ‘You best understand and appreciate your own health care system by analysing other health care systems,’ the answers to the above questions and the discussion can provide valuable insights for health policy-makers in other countries.

## Regulatory framework in Belgium and the Netherlands

We are using a broad definition of health technology, including implants, prostheses, in vitro diagnostics and drugs, as well as equipment. Table 2 provides health technology examples that in 2015 were not covered by mandatory basic health insurance in Belgium.

**Table 2.** Health technology examples: situation in Belgium and the Netherlands (2015) (same examples as Table 1).

Technology	Belgium	The Netherlands
Robot-assisted cardiac surgery A form of heart surgery performed through very small incisions in the chest. With the use of tiny instruments and robotic devices, surgeons are able to perform several types of heart surgery in a way that is much less invasive than other types of heart surgery. The procedure is sometimes called da Vinci surgery because that is the name of the robot often used for this procedure.	Not covered by mandatory basic health insurance. The cost is to be borne by the patient.	Covered by mandatory basic health insurance. Physicians, hospitals and insurers choose whether, where and when robot-assisted cardiac surgery can be used.
Trabecular metal acetabular revision system (TMARS) Hip revision surgery involves the removal of failed implants, and replaces them with new ones. The use of trabecular metal increases implant stability and enables biologic in-growth, which can help lead to long-term fixation.	Not covered by mandatory basic health insurance until 2014. The cost was to be borne by the patient. As from 2014, TMARS is covered by mandatory basic health insurance.	Covered by mandatory basic health insurance. Physicians, hospitals and insurers choose whether, where and when TMARS can be used.
Cervical intervertebral disc prosthesis (e.g. MOBI-C, Bryan Cervical Disc) An alternative for cervical spinal fusion for the treatment of symptomatic (e.g. radicular neck and/or arm pain and/or functional/neurological deficit) cervical degenerative disc disease or herniated disc. The cervical intervertebral disc prosthesis is authorized by the American Food and Drug Administration (FDA) [9], acknowledged by health technology assessment centres (e.g. National Institute for Clinical Excellence [10]) and is being covered by leading American health insurers (e.g. Blue Cross /Blue Shield [11]).	Not covered by mandatory basic health insurance. The cost is to be borne by the patient.	Not covered by mandatory basic health insurance. The prosthesis does not meet the criteria of ‘current scientific knowledge and practice’ in the Netherlands [12]. The prosthesis cannot be separately billed to the patient.
Microprocessor-controlled prosthetic leg (e.g. C-Leg, Genium) A prosthetic limb with several sensors that gather and calculate data. These computerized prostheses are claimed to be a significant improvement over the conventional mechanically controlled prostheses.	Not covered by mandatory basic health insurance. The cost is to be borne by the patient.	This prosthesis is rarely covered by mandatory basic health insurance, only when the mechanical alternatives do not provide an adequate solution.
MammaPrint A gene assay that may help to identify those breast cancer patients that may safely forgo chemotherapy.	Not covered by mandatory basic health insurance. The cost is to be borne by the patient.	Although the National Health Care Institute (‘Zorginstituut Nederland’) has stated that the MammaPrint does not meet the criteria of ‘current scientific knowledge and practice’ [13], many Dutch health insurers do provide coverage.
Non-invasive prenatal genetic testing (NIPT) A serum marker screening for certain chromosomal abnormalities in a developing foetus (e.g. Down syndrome).	Not covered by mandatory basic health insurance. The cost is to be borne by the patient.	Not covered by mandatory basic health insurance. NIPT is available only in 8 university hospitals in the context of study protocols Trident 1 and 2. As from 1 April 2014 until 31 March 2017 there was only access for pregnant women presenting a high risk for a trisomy baby (Trident 1) [14]. As from 1 April 2017 all pregnant women will have access to NIPT in the context of the Trident 2 study [15].
Ofatumumab (Arzerra) An orphan medicine used to treat chronic lymphocytic leukaemia.	Not covered by mandatory basic health insurance. The cost is to be borne by the patient.	Covered by mandatory basic health insurance.
Nivolumab (Opdivo) A cancer medicine used to treat adults with melanoma or squamous non-small cell lung cancer.	Not covered by mandatory basic health insurance in 2015 In principle, the cost is to be borne by the patient. However, a ‘compassionate use’ /‘medical need’ programme was running for Nivolumab in 2015. As from 1 April 2016 Nivolumab is covered by mandatory basic health insurance.	Not covered by mandatory basic health insurance in 2015. The Dutch Health Authority has stated that the use of Nivolumab was not cost-effective [16]. As from 1 March 2016 Nivolumab is covered. by mandatory basic health insurance because, after negotiations, the price has been reduced [17].

### Belgium

Health insurance in Belgium operates as a reimbursement system. All goods and services that are covered by mandatory basic health insurance have a six-digit code. In the case of a life-threatening or rare disease, an intervention from a ‘Special Solidarity Fund’ (‘Bijzonder solidariteitsfonds’, a public fund) can be asked for products that are not (yet) covered by basic health insurance [18]. This fund decides on a case per case basis about reimbursement for individual patients.

Implants need to be notified to the National Institute for Health and Disability Insurance (‘Rijksinstituut voor ziekte- en invaliditeitsverzekering’). However, notification does not automatically imply reimbursement by basic health insurance (with reimbursement rates varying between 100% and 12%). In case costs of the application procedure exceed foreseeable profits, medical firms may not apply for reimbursement, e.g. in case of new technology that will be only rarely used. For notified implants that are not covered by basic health insurance the full cost has to be borne by the patient.

The template of the hospital bill is defined by law. Drugs, implants, prostheses and other medical devices that are not covered by mandatory basic health insurance have to be explicitly mentioned on the bill. Some hospitals provide extensive lists of non-covered goods and services that need to be paid for out-of-pocket [1,2].

In every hospital there is a ‘Committee for Medical Material’ (‘Comité voor medisch materiaal’), where management, pharmacists and physicians sit together to discuss what medical material can be used within the hospital. The committee for medical material creates and updates a formulary of medical material being used in the hospital. Health technology and health economics assessments are being made. The most important criteria are patient safety, added value (compared to similar products) and cost. There are three options: reimbursement by mandatory basic health insurance (or by the ‘Special Solidarity Fund’), financing by the hospital (e.g. a special fund created by the hospital) or billing to the patient.

Non-coverage is common for health technologies for which there is no (complete) unanimity (yet) about the evidence. For example, although cervical intervertebral disc prostheses are approved by the American FDA, there is no unanimity about the use of these prostheses. In Belgium, lumbar intervertebral disc prostheses are reimbursed by basic health insurance, whereas cervical prostheses are not. American health insurance companies such as Aetna and Blue Cross/Blue Shield do reimburse cervical prostheses but not lumbar prostheses.

Eighty-two per cent of all Belgians benefit from voluntary additional hospital insurance (figure for 2015) [19,20]. This additional insurance is covering co-payments, supplementary physician’s fees and health technology that is not (yet) reimbursed by basic health insurance. The coverage for health technology constitutes an important element for competition between insurance companies providing additional health insurance. Coverage for new medical devices and drugs differs strongly from company to company.

According to the Belgian Patient Rights Act, health care providers are obliged to inform their patients about the different treatment options and the cost for the patient [21]. However, physicians may be reluctant to do so (and thereby raise hope) if they expect that the patient eventually might not be able to pay out-of-pocket for the new implant or the new drug [22].

### The Netherlands

In the Netherlands, there is a mandatory basic health insurance. On top of this mandatory insurance, 84% of the Dutch have subscribed to voluntary additional health insurance (figure for 2015) [23].

When assessing out-of-pocket payments for new health technology in the Dutch health care system, three elements have to be taken into consideration.

1. How are entitlements to care defined within mandatory health insurance? In Belgium there is a clear list of medical goods and services that are covered by mandatory insurance. Entitlements to care are explicitly formulated. As a consequence, it also clear which care is not covered (= care which is not on the list). In the Dutch regulation there is a non-explicit, open formulation of the entitlement to care. Dutch law stipulates that the insured is entitled to care which conforms with the criteria of ‘current scientific knowledge and practice’ (Dutch: ‘stand van wetenschap en praktijk’). It is the individual insurer that in first instance decides, by contracting with individual hospitals, which specific treatments are effectively available for their insured. The National Health Care Institute (‘Zorginstituut Nederland’) checks some new technologies for their conformity with the criteria of ‘current scientific knowledge and practice’, e.g. very expensive technologies. In theory, all care which is in conformity with the criteria of ‘current scientific knowledge and practice’ is reimbursable. However, in practice the possibilities for the application and the reimbursement of new technology are not unlimited, and choices are being made by insurers and providers. These choices are being reflected in the contracts between insurers and hospitals. Budgetary considerations play a role in the choices made (7 May 2015 email from J Hallie, Zorginstituut Nederland; unreferenced). Consequently, specific goods and services that are covered by mandatory health insurance, may appear not to be available in clinical practice, as a consequence of specific budgettary restraints or other elements in the contract between the insurer and the hospital.
2. According to article 35, §1 of the Health Care Market Regulation Act (‘Wet Marktordening Gezondheidszorg’), health care providers are allowed to charge only a global price for a ‘Diagnosis Treatment Combination’ (‘DTC’). A DTC comprises all inpatient and outpatient treatments for a certain diagnosis during a certain period of time, e.g. three months (in Dutch: ‘Diagnose Behandel Combinatie’, ‘DBC’). A consequence of this ‘integral tariff system’ is that all costs of the treatment trajectory have to be included in the tariff.
3. The distinction between a benefits-in-kind policy (‘naturapolis’) and a reimbursement policy (‘restitutiepolis’) is important. Unlike Belgium, where there are only reimbursement policies, Dutch insured can choose between a benefits-in-kind policy and a reimbursement policy. In the Netherlands in 2015, 55% of the insured had a benefits-in-kind policy, 23% a reimbursement policy and 22% a combined policy (benefits-in-kind for some types of care and reimbursement for other types of care) [24].

With a benefits-in-kind policy, the patient gets the treatment that has been bought by the insurer from the contracted provider. Whether this treatment includes new health technology depends on which treatment has been bought by the insurer. A benefits-in-kind policy entitles the insured to receive care and obliges the insurer to deliver or contract the care (‘duty of care’; in Dutch: *zorgplicht*). If the insured visits contracted providers, the insurer pays the full bill to the provider. If the insured visits a non-contracted provider, the insured receives from the insurer a reimbursement as determined in the insurance contract (e.g. 75% of the usual price in the market). Anyway, because of the integral tariff system, no supplement can be charged by the provider to the patient for the use of new health technology. The insurer and the hospital have the contractual freedom not to include expensive new health technologies, although they meet the criteria of ‘current scientific knowledge and practice’ (17 June 2015 email from K Siemeling, Zorginstituut Nederland; unreferenced; 18 June 2015 email from JP Plass, Nederlandse Vereniging van Ziekenhuizen; unreferenced). They could choose for a cheaper or more cost-effective substitute that also meets the criteria of ‘current scientific knowledge and practice’. For example, an insured patient who needs prostate surgery is entitled to receive this surgery, but whether or not it is robot-assisted surgery depends on the care that the insurer has purchased from the hospital to which the patient is admitted. Because of his duty-of-care (in Dutch: ‘zorgplicht’), an insurer must always make sure that his insured are receiving the appropriate care that they are entitled to and that meets the criteria of ‘current scientific knowledge and practice’. An important question, then, is in how far there is transparency for the insured about the use of new health technology?

A reimbursement policy entitles the insured to being reimbursed for his health care expenses, insofar as the prices charged are market conform. The patient, as the purchaser of care, is concluding a contract with the health care provider. In principle, the provider could bill the full price to the patient, including a ‘supplement’ for new health technology (as part of the integral tariff), and the health insurer could limit reimbursement to the market-conform price, which might be lower than the price charged by the provider. In order to find out whether this possibility is effectively being applied, we have contacted the association of hospitals (‘Nederlandse Vereniging van Ziekenhuizen’), the association of medical specialists (‘Federatie Medisch Specialisten’), the association of health insurers (‘Zorgverzekeraars Nederland’), as well as the National Health Care Institute (‘Zorginstituut Nederland’). Based on their answers, there is no doubt that the general perception is that in Dutch hospitals in practice no such ‘supplements’ on top of the regular ‘DTC price’ are being charged (7 July 2015 email from ACM Van Harderwijk, Federatie Medisch Specialisten; unreferenced; 18 June 2015 email from JP Plass, Nederlandse Vereniging van Ziekenhuizen; unreferenced; 4 and 8 June 2015 emails from J Veerkamp, Zorgverzekeraars Nederland; unreferenced; 17 June 2015 email from K Siemeling, Zorginstituut Nederland; unreferenced).

In the Netherlands, a new treatment may be conditionally accepted, when its ‘cost’-effectiveness still has to be proven. This new treatment is then being offered in a limited number of hospitals only. For example, from 1 April 2015 until 1 October 2019, hyperthermic intraperitoneal chemotherapy (HIPEC) for the treatment of peritoneal carcinosis for patients with colorectal cancer is being provided in seven hospitals [25]. After 1 October 2019, a decision will be taken whether or not HIPEC will be reimbursed by mandatory basic health insurance.

So far as prostheses are concerned in the Netherlands, reimbursement is limited to the cheapest adequate solution.

When basic health insurance does not (yet) reimburse, both in Belgium and the Netherlands, medical firms sometimes set up a compassionate use/medical need programme, whereby medical firms finance the cost of new drugs or new medical material.

## Conclusion

The Dutch health care system is very much an egalitarian system. Expenditure on general hospitals is almost completely covered by mandatory basic health insurance. Out-of-pocket expenditure represents only 0.4% of total expenditure on hospitals. The situation is very different in Belgium, where private expenditure on general hospitals amounts to 17.5% of total expenditure on hospitals. Additional health insurance is covering 8.5% of total expenditure on hospitals [26]. In 2015, 9.2 million Belgians benefited from voluntary additional health insurance (82% of the population) [19,20].

While in the Netherlands it is theoretically possible to charge a supplement for new health technology to patients who have a reimbursement policy, the general perception is that this is not happening in practice. Although the Dutch government is promoting competition on price and quality among health insurers and health insurance policies and although legislation allows them to do so, health insurers are not offering two benefits-in-kind policies A and B whereby for policy A treatment A has been contracted and for – the more expensive – policy B treatment B, using new health technology, has been contracted.

Recently, concerns have been raised in the press about Dutch hospitals not always or not immediately providing the patient with the best treatment available for financial reasons. For example, bevacizumab (Avastin) might not be given to all patients with colon carcinoma because some hospitals prefer not to pay for this expensive treatment [27].

Contrary to the Netherlands, Belgium has a two-tiered system so far as access to new health technology is concerned. Access to new health technology depends on the patient being informed about the new technology and the ability and willingness to pay out-of-pocket. Covering new health technology that is not (yet) reimbursed by basic health insurance is one of the reasons for the existence of additional health insurance in Belgium.

### Access to new health technology in the Netherlands

Certain treatments are covered for nobody by mandatory health insurance in the Netherlands, e.g. cervical intervertebral disc prosthesis (see Table 2). These treatments may also not be charged to the patient. As a consequence, they are not accessible for Dutch patients. Other treatments are covered, but under strict conditions, e.g. non-invasive prenatal testing (see Table 2). Patients who do not meet the conditions may be tempted to look for these treatments abroad. Often, new technologies are covered by mandatory health insurance in the Netherlands that are not covered in Belgium (see Table 2). However, it is not always clear for the Dutch patient which insurers and which hospitals do offer a specific new health technology. In principle, the patient can check the website of the insurer or enquire with the insurer by telephone. Insurers are obliged to give a detailed answer to such questions. However, in practice this possibility is not often used.

### Explanation of observed differences

The regulatory framework is an important explanatory factor for the differences between Belgium and the Netherlands. In Belgium there is a closed, enumerative list of medical goods and services covered by mandatory health insurance. As a consequence there is transparency about which treatments are not being covered. In the Netherlands there is no such list. Dutch law stipulates that care that meets the criteria of ‘current scientific knowledge and practice’ is to be covered by mandatory health insurance. However, unless the National Health Care Institute has assessed a certain treatment, insurers and hospitals do not necessarily all have the same approach towards that treatment (22 May 2015 email from J Hallie, Zorginstituut Nederland; unreferenced). This may cause a less transparent situation for the patient in the Netherlands.

The existance of in-kind health insurance policies in the Netherlands, as opposed to Belgium, may also help explain differences in access to new health technology. With in-kind policies, patients’ choice is limited to the care contracted by the health insurer.

### Consequences for the patient

Allowing out-of-pocket payments (or coverage by additional health insurance) for new health technology of course has consequences for the patient (see Table 3). Whereas the Belgian approach may do better in terms of ‘access to new health technology’ for those who are able and willing to pay, the Dutch approach has a better score for ‘equal access to care’. In Belgium, patients have more choice, if they can pay. Of course, condition is that they are informed about the existence of other treatment options. Based on the Patient Rights Act of 2002, their doctor should inform them about all treatment options, including those that are not covered by mandatory basic health insurance. More research is needed on the question of to what extent doctors effectively perform this task. For instance, doctors might be inclined to only inform well-off patients who can afford to pay out-of-pocket for an expensive new health technology.

**Table 3.** Effects of allowing out-of-pocket payments for new health technology.

Criteria	Effect
Access to new health technology for those who are able and willing to pay	Positive
Equal access to care	Negative
Choice	Positive
Transparency	Positive

Within the Dutch health system there is less transparency on the availability of new health technology. Out-of-pocket payments for new health technology do not exist in the Netherlands. The comprehensiveness of the statutory benefits package may be part of the explanation. However, since it is impossible for the benefits package to cover all new health technologies, Dutch patients may not have access to certain new technologies. It is quite likely that some patients may go abroad in order to get access to these technologies by paying out-of-pocket.

Yearly, about 2,500 Dutch patients who do not meet the conditions for reimbursement of the non-invasive prenatal test (NIPT), have the test performed in Belgium [28]. In its letter of 13 January 2015 to the Dutch parliament, the Dutch government stated that no official data are available about physicians in the Netherlands referring pregnant women to hospitals in Belgium or sending blood samples to laboratories abroad for a NIPT. The government stated that in the Netherlands the NIPT can only legally be performed in the context of a study protocol and that physicians who collaborate with laboratories abroad might be breaking the law [28].

Another element of the Dutch health care system may also negatively affect transparency. Since health insurers and hospitals are free to contract, including on the use of new health technologies, the patient may not know about new technologies being used in one hospital but not in the other.

There seems to be a trade-off between equal access to care on the one hand and choice and transparency on the other. In a two-tier health care system, there is no equal access to new health technology. In an egalitarian system transparency on where and what technology is being used, as well as choice are limited.

Eighty-two per cent of the Belgian population and 84% of the Dutch population has subscribed to additional health insurance (figures for 2015) [19,20,23]. While Belgian additional insurance is mainly offering coverage for inpatient costs, Dutch additional insurance focuses on outpatient costs such as dental care and physiotherapy. As opposed to Belgium, additional health insurance in the Netherlands does not offer coverage for new health technology which is not (yet) covered by basic insurance. In Belgium, the role of additional insurance in covering new health technology is recognized by the government. The Belgian ‘Special Solidarity Fund’, which is an integral part of mandatory basic health insurance, explicitly stipulates that patients first have to seek reimbursement for a new technique from their voluntary additional health insurance before they can file a request with the Fund [29].

## Discussion

Comparing access to new health technology in Belgium and in the Netherlands, two neighbouring countries, leads to some interesting discussion points. What are the consequences of a more egalitarian versus a more libertarian approach? What are the consequences for the patient of open non-explicit versus closed explicit description of entitlements? What is the role of voluntary additional health insurance in providing access to new health technology? And what are the health policy implications for other countries?

### Egalitarianism versus libertarianism

In 2008, a review commissioned by the British government was published by Richards on how patients might combine privately purchased care with care provided by the National Health System [30]. Richards [30] sees a tension between the principle of equity and the principle of personal autonomy. The term ‘equity’ is used in a broad sense to mean that every person should have access to health care on the basis of need and not ability to pay [31]. The term ‘autonomy’ is used to denote a very specific principle, namely people’s right to spend their money as they choose. One could argue that a health care system could meet both the equity and the autonomy principle by offering a comprehensive basic health insurance on the one hand and the individual right to buy health technology that is not (yet) covered by basic health insurance on the other hand. The tension between equity and autonomy is being reflected by two opposite views on the provision of health care: the libertarian and the egalitarian view [32]. In the libertarian view, access to health care is part of society’s reward system, and, at the margin at least, people should be able to use their income and wealth to get more or better health care than their fellow citizens should they so wish. In the egalitarian view, access to health care is every citizen’s right (like access to the ballot box or to safe drinking water), and this ought not to be influenced by income or wealth.

Although the Dutch decentralized system with competing insurers allows for, in theory, the insurers to offer health insurance products that compete on price and quality, we concluded in the previous paragraph that in practice we do not observe competing health insurance products that distinguish themselves by offering access to the latest new (expensive) health technology. Although there are some differences among the competing health insurance products offered in the Netherlands, these differences are not related to new (expensive) health technology. Therefore, as far as access to new health technology is concerned, the Netherlands in practice seem to favour a more egalitarian approach, while the Belgian approach may be perceived as more libertarian.

### Open non-explicit versus closed explicit description of entitlements

In many countries there is a strong tendency towards greater transparency about the quality of care. With a clear, closed list, entitlements to care tend to be transparent and explicit, as opposed to a system with an open, non-explicit description of entitlements. Implicit and non-transparent entitlements can be illustrated by Kaiser Permanente generally defining ‘a covered service’ as ‘one performed or prescribed by a Permanente doctor’ [33]. But what are the consequences of greater transparency on new health technology for an egalitarian health care system? A greater transparency might reveal the existence of inequalities in access to new health technology, e.g. new technology not being covered by basic insurance or only for certain groups. Of course, such inequalities are at odds with the premises of an egalitarian system.

### Voluntary additional health insurance

Voluntary additional health insurance can offer coverage for health technology that is not (yet) covered by mandatory health insurance.

Coverage by additional health insurance can be limited, with only certain types of health technology being covered or caps being applied, but coverage can also be more extensive. Coverage of new health technology can be a major competitive factor among additional health insurers. This is, for instance, the case in Belgium. This competition among additional health insurers is in line with Pauly’s pleading for competition among health plans based on the rate at which new technology is introduced [34]. In a highly standardized market for health insurance, any additional treatment or drug covered by an insurance contract may be a decisive factor encouraging patients to sign it with the insurer offering the widest or most differentiated coverage [35].

Access to new health technology may also be influenced by the interaction between mandatory basic insurance and voluntary additional insurance. Mandatory health insurance can decide not to cover a certain medical technique and to wait for new evidence or for prices to decrease. In the meantime the technique can be financed out-of-pocket or through additional insurance. Out-of-pocket financing and additional insurance can play the role of a ‘waiting room’ for promising new health technologies, before they are being covered by mandatory insurance. Of course, this will only work for technology for which there is sufficient (and growing) evidence. For example, as from 2011, the TMARS hip prosthesis (see Tables 1 and 2) has been covered by additional health insurance in Belgium before being covered by mandatory health insurance as from 2014. Mandatory health insurance taking over coverage from additional insurance can free up financial resources with additional insurers, allowing them to finance other new technologies. Since access to voluntary health insurance may be difficult for ‘the sick, the old and the poor’, the ‘waiting room’ function of additional insurance should be limited and mandatory health insurance should be offering a comprehensive coverage of new health technology.

### Policy implications for other countries

Accessibility of new health technology which is not (yet) reimbursed by mandatory basic health insurance is an important health policy issue. The reason a new health technology is not covered by mandatory insurance can be the lack of unanimity on its evidence-based character. A technology may be successfully assessed and reimbursed in one country, but not in another one.

Prohibiting access to new health technology which is not (yet) covered by mandatory insurance may prove to be difficult to enforce. Rather, in order to protect citizens from paying out-of-pocket for totally ineffective technology, information can be provided on the reasons why some new health technology has not been included in the mandatory benefits package. Mandatory registration of all new health technology can be used to prevent unsafe health technology from being marketed and used.

The availability of clear information on new health technology that is not (yet) reimbursed by mandatory basic health insurance is a crucial factor. In a globalizing world, such information is likely to be increasingly available, at least for people that are well networked.

An analysis of the Belgian and the Dutch approach reveals that a closed explicit system of entitlements to care may create an environment in which patients (and their doctors) are encouraged to look for and to use new health technologies which are not (yet) reimbursed by mandatory insurance. Reimbursement by additional health insurance can also facilitate the use of new health technologies, e.g. by providing reimbursement for technologies that are not yet reimbursed by mandatory basic health insurance but that are under review for reimbursement (= ‘waiting room function’). Risk-averse individuals may want to protect both their health and their wealth by assuring access to expensive health technology not (yet) covered by mandatory basic health insurance. In all types of health systems, there is an increasingly concerted effort to specify explicitly an ‘essential’ package of health care that is covered by mandatory health insurance [36]. Because of increasing offer and demand of health technology and growing budgetary constraints, the comprehensiveness of the mandatory package of care is coming under strain. Smith [37]S has investigated the question of how to choose the mandatory package to which all citizens are given free access when objectives include financial protection as well as health improvement. A key concern is the type of private markets available and the nature of patients’ responses when a treatment is not covered by such a package. Smith [37] has modelled three scenarios: no availability of private care; a spot market of private care paid for out-of-pocket; and a market in prepaid complementary private insurance. His conclusion is that governments can secure an optimal system of mandatory health insurance coverage by specifying a benefits package in line with redistributional goals and nurturing a complementary voluntary insurance market [37]. He argues that under these circumstances, conventional cost-effectiveness analysis is the appropriate decision rule for including treatments in the package. Certainly, more research is needed on the interaction between cost-effectiveness analysis and insurance design [38].

## Notes

No funding was received for the manuscript.

The research has not been sponsored.
